# The New York Institute of Stomatology

**Published:** 1905-03

**Authors:** 

**Affiliations:** The New York Institute of Stomatology


### Reports of Society Meetings.

### THE NEW YORK INSTITUTE OF STOMATOLOGY.

A MEETING of the Institute was held on Tuesday evening, December 6, 1904, at the "Chelsea," No. 222 West Twenty-third Street, New York, the President, Dr. C. O. Kimball, in the chair.

The minutes of the last meeting were read and approved.

Dr. Lee Maidment Hurd read a paper entitled "The Radical Treatment of the Maxillary Antrum."

(For Dr. Hurd's paper, see page 159.)

### DISCUSSION.

*Dr. T. Passmore Berens.*—Dr. Hurd's paper has been such that it leaves but little to be added. However, the subject is one of considerable interest and of such importance that perhaps you will pardon my emphasizing one or two points in the doctor's remarks.

In discussing the subject before us,—chronic empyema of the maxillary antrum,—it is necessary to understand what is meant by chronic suppuration of this cavity. Chronic empyema may be defined as a suppuration that has persisted more than six months, although, as the writer has stated, it may apparently begin as a chronic disease.

The etiology of these cases frequently is remote, but in the majority of instances the infection may be traced to various intra-nasal infections,—*e.g.*, grippe, scarlet fever, etc.,—or to a direct infection by diseased teeth.

The first class of infections of the antrum, those of nasal origin, usually are associated with a more or less extensive infection of all or part of the pneumatic cellular structure of the rest of the accessory sinuses,—viz., the ethmoid cells and the sphenoid sinus; and in many of these cases the maxillary antrum acts only in a passive way as a reservoir for the pus formed in the superimposed ethmoid cells. This pus, in turn, sets up various pathological changes in the lining of the antrum, and usually results in granulations and myxomatous tissues. These changes in the antrum may, and most frequently do, cause various symptoms in the teeth, the most frequent of these being dentalgia. In many of my cases this dentalgia was the most prominent symptom present, and the particular symptom from which the patients sought relief, a relief often too readily but vainly promised by extraction.

A diseased tooth may be, and not infrequently is, the cause of antrum disease, usually of an acute type; and, unless prevented by the proper treatment, this type may rapidly change into a chronic one. Given a tooth with an ulcerated root protruding into the antrum, and allowed to remain until granulations and necrosis have formed about it before its extraction, obviously the extraction of the tooth *per se* may not remove the necrosis and granulations. The necrosis may be extensive and the granulations form a large mass in the cavity of the antrum, and while the extraction permits of drainage through the tooth socket, this drainage frequently is insufficient to control the spread of the necrosis or the growth of the granulations, and we thus have the beginning of a stubborn antrum disease; again, the incomplete extraction of diseased roots will result in the same condition.

I have seen several instances of each of these conditions. In cases of this character there may readily be an extension of the disease into the other accessory sinuses. A point I wish to emphasize is that a tooth that has been troublesome for a long time,—several months,—if it has caused antrum disease at all, may have caused empyema long before the empyema is suspected, and thus the condition may really be a chronic empyema from a tooth when first it is diagnosed; and, therefore, that a discharge that does not yield readily to treatment after tooth extraction and proper treatment through a drainage opening should be suspected to belong to this class of chronic cases and treated accordingly. Another point I wish to make is that cases of dentalgia accompanied or not by other neuralgias, or by nasal pus, should be suspected as of antral origin, even when in the presence of decayed teeth; that is, that antrum disease and not the decayed teeth may be causing the dentalgia.

To be clearly understood, briefly stated, I believe that antrum disease is caused many times by diseased teeth; but, on the other hand, that many a sound tooth has been sacrificed when proper treatment of the antrum would have saved it. I believe also that extensive disease in the antrum may under certain circumstances cause ulceration and death of a tooth.

During the last two years I have operated on eighteen cases of chronic empyema of the maxillary antrum. Fifteen of these cases were successfully operated upon by the Jansen operation, similar to the operation described by the author. Three cases were operated upon by milder measures. Of all of these cases, twelve had a history of some sort of trouble with the teeth; in four, diseased roots were found on operation lying buried in granulation tissue, and necrosis in the floor of the antrum. In three other cases all of the teeth of the upper jaws had been wrongly extracted years before for cure of the dentalgia; and in one case the teeth were saved from extraction and the dentalgia cured by operative measures.

As to the operation of preference in these cases, there is no rule that can be laid down for our guidance; we must be guided by our general experience, together with a careful study of each case. In advanced cases, and especially when multiple sinusitis is present, as it practically always is, the operation of Jansen, as described so accurately by the reader of the paper, is beyond question, in my hands, the most satisfactory. In less advanced cases the Moure or Caldwell-Luc is sufficient.

In any operation for chronic suppuration, however, it is a *sine qua non* of success that a large free permanent opening be made at the floor of the antrum and between it and the nose. If this opening is sufficiently large and far forward, with proper appliances a good view of the antrum may be had and the result of its treatment noted.

Dr. Dawbarn wished to congratulate Dr. Hurd upon his excellent operative results in a class of troubles where good results and not persistence of lingering annoyance from discharges, etc., means that thorough work has been done. Dr. Dawbarn proceeded to discuss several points of the paper. Alluding to the fact that frontal sinus suppuration is sometimes a cause of antrum infection, and not always suspected where the infundibulum is large and so pus drains freely and without its retention causing frontal pain, he mentioned that the opening of the frontal sinus and that of the antrum lie side by side, the former anterior, in the middle meatus of the nose; so that in dorsal recumbency the frontal pus necessarily drains directly across the antrum opening, or, more properly, openings; for though there is but one in the skeleton head, there generally are two, quite small and close together, in the mucous membrane here.

Dr. Hurd had taken the position, in his address, of believing that disease of the roots of teeth in the antral floor is a very common cause of disease of this cavity. In this he is widely at variance with numerous careful observers, though admittedly he can find others agreeing with him. But in a paper on this disease,[1] Dr. Dawbarn quoted Dr. Stout, of Philadelphia: "Dmochowski, after making one hundred and fifty autopsies (for this purpose), holds that but few cases have a dental origin." Again: "Fletcher, in five hundred skulls, in two hundred and fifty-two of which there were abscesses of the upper molars, found only twelve molars perforating the floor of the antrum." Also: "Carious teeth would appear to be an effect rather than a cause of antritis (Fränkl)." Stout himself states that he has never seen a case caused by carious teeth. However, Mears, of Philadelphia, agrees with Dr. Hurd in thinking the teeth the chief cause.

Dr. Dawbarn called attention to the very severe operations necessary in the cases shown to-night, and said that doubtless Dr. Hurd had not intended to indicate, by not mentioning the mild plans of treatment used in mild cases of antrum disease, that he treated all cases in this manner. On the contrary, a free opening in the anterior wall just above the gum, permitting admission of the little finger, and thus enabling a careful study of the cavity, so differing in different people, and in simple cases draining this and gently packing with mild, soothing antiseptic gauze—this plan

[1] "Disease of the Antrum," read before the Central Dental Association of Northern New Jersey, April 16, 1900, published in Items of Interest.

suffices for many cases, gradually to effect a cure. But in the really severe cases, and also those which had become chronic through years of neglect, we are face to face with a problem practically identical in principle with that met by the surgeon in empyema of the pleural cavity,—namely, a rigid walled cavity lined with a membrane discharging pus into that cavity. And where the lung is bound down by inflammatory exudate, mere drainage of that pleural space by excising an inch or two of a rib, low down, to permit of good drainage through tubes would almost never suffice. Either the outer wall must be made to collapse against the inner by dividing a series of ribs, or (as recently is being done) at the same operation dividing, and even stripping off sometimes, the thick, unyielding membrane tightly holding the collapsed lung, this organ quickly expands to meet the ribs; and then a cure follows by "secondary adhesion," the growing together of the two surfaces which so long have formed pus. Now, in the antrum, similarly, given the worst inflammatory diseases of it, we must, as Dr. Hurd's cases show, carry out the same idea, or else expect a permanent continuance of the trouble. As it is not feasible here to remove the rear wall nor the upper, and not, as a rule, the inner (unless these are themselves diseased), there remains the front bony wall; and this is most freely sacrificed, together with the suppurating lining membrane. Healthy flesh fills in the space thus left, and with much less evidence of the operation left than would be supposed. All cutting through the skin can generally be avoided, in such operations, by those experienced in such work; thereby much adding to the patient's gratitude.

*Dr. Fossume.*—Dr. Hurd's paper is most instructive, and I would like to relate the circumstances of a case that I had under treatment last spring, as we stomatologists are most interested naturally in cases which are in line with our actual professional work. The patient had been suffering a good many years with a continuous pain in the upper right side in the vicinity of the molars. There was distinct heaviness, together with some orbital and frontal pain. The two molars and the first bicuspid were loose, and directly above and between the buccal roots of the first and second molars there was a discharging fistula. She objected very much to losing the teeth. I opened the three teeth and found the pulp of the second bicuspid congested and semivital, and this I extirpated by pressure anæsthesia, and sterilized the canal and filled it. The roots of the molars were partly filled but badly infected, and there was a very distinct putrescent odor. The canals in all six roots were very small, and it was with great difficulty that I cleansed and filled them. As the patient had not the slightest relief after the canals were filled, and the discharge had not abated in the least, I injected beta-eucaine hypodermically around the buccal roots of the molars, and with a long shank bur bored away the alveolus and amputated the ends of the posterior buccal root of the sixth-year molar and the anterior buccal root of the twelfth-year molar. I found these roots penetrating the antrum, and although I do not really believe the case was one of true abscess of the antrum, I feel positive that the lining membrane was affected.

When I syringed the wound, the solution would pass through the nose. In the dressing and the after-treatment of such a case I have found the greatest help and most satisfactory results from using carbolized oil (a solution of 1 in 40), and a little hydronaphthol. I have substituted at times boiled glycerin and aristol, but have always had to go back to the carbolized oil on account of its soothing influence on the tissues, stopping pain at once. Furthermore the dressing will never adhere to the wound, but comes away readily.

This case was discharged as cured in three weeks, the teeth became perfectly firm, and the abscess disappeared. I have had occasion to see the case this winter, and the teeth are in perfect condition.

*Dr. Leo Green.*—I am at a loss to understand why the old operation has been so universally condemned to-night as being useless. It has been my fortune to come in contact with several nose and throat men, and I have seen them perform operations in this manner with success. There need be no danger of food getting into the opening during mastication. To prevent this I take an impression of the opening immediately after the operation, with a piece of orange-wood stick, and from this impression make a hard rubber plug to fit the opening, having a shoulder below. This can be removed whenever necessary, to syringe the antrum. I have followed up a number of these cases, other than those upon which I operated, with uniformly good results. I have in mind one case which seemed to call for a radical operation, but to which I was opposed. It was my opinion that the trouble in this case was due to an injury to the nerve in the anterior buccal root of the molar, caused by the bur in drilling into the antrum. The tooth was finally extracted, and this condition was found to obtain. The indications for a radical operation disappeared after the extraction.

*Dr. F. Milton Smith.*—I do not understand that Dr. Hurd advises these operations unless in extreme cases of antrum trouble where all other measures of a milder sort have failed. My own experience in this line of work has been very limited. I have seen, in my practice of thirty years, four or five cases which in one way or another involved the antrum. I have read some on the subject, talked with those who know of the matter, and have the impression that in years gone by some of our leading men along these lines have taught us that about the best we could do with these old chronic cases was to keep them in as cleanly a state as possible. Of course, the operation Dr. Hurd has described to us to-night is radical, and ought not to be done unless it is needed, but it has seemed to me that as between wearing one of these drainage-tubes all my life and submitting to such an operation, I should prefer the latter.

Just a word regarding some of these simple cases that do not come within the range of Dr. Hurd's operation, where there is possibly simply a puncture into the antrum from an abscessed root or some other cause. I had under my observation some ten years ago a patient in one of whose upper molar teeth the pulp in one of the canals had become devitalized. It had gone on to suppuration, causing a discharge of pus into the antrum. He had called upon a dentist, who had opened the tooth and then sealed it up again and gone away from town. During the week of his dentist's absence he came into my office suffering terrific pain. I could not account for the acute pain until the tooth was opened, when I found live pulp-tissue in the remaining two roots, the extreme pain coming from the pressure of the pus upon this sensitive tissue. After evacuating the pus I treated the canals once or twice and then filled the roots as I would fill an ordinary root. Then through an external opening I treated the antrum a number of times, until one day he appeared for treatment and I was unable to find any opening. It had healed. I have no doubt that if I had not treated as long as I did it would have healed more quickly. This seemed to be illustrated by another case that I had where there was a discharge into the antrum from a suppurating condition of the roots of a tooth. As it was a tooth that had given considerable trouble, I decided to have it out, and after the extraction thought I would see what would happen without any treatment of the antrum at all. The case got entirely well of its own accord.

Two or three years ago a patient had trouble with his antrum. His dentist told him the trouble was caused by the root of a tooth left in after an extraction. He had advised that the antrum be opened and thoroughly treated. He was not willing to have this done, and eventually fell into my hands. I told him that perhaps a mild antiseptic used with the nasal douche might be all that was necessary. He used this for about a week and came back vastly improved. In three weeks' time there was no further disturbance. I mention these cases merely to show that there are cases that do not need operative treatment, and for such cases I am confident Dr. Hurd does not advise the radical operation.

*Dr. Hurd.*—I believe I stated in my paper that it did not have reference to simple cases. Nearly all of the acute cases will get well without an operation. Of the chronic cases, some will get well and some will not, and it is in these latter cases that we perform the radical operations.

With regard to the walls of the antrum collapsing, I do not see how the anterior wall of the antrum can collapse much.

In regard to transillumination as a test for disease of the antrum, sometimes it fails entirely. In the same mouth I have seen the normal side appear denser than the affected side.

Dr. J. W. Canaday, of Albany, read a paper entitled "A Study of the Dental Club in its Relation to the Dental Society."

*The President.*—I feel that the great value in the paper will be found, not so much in relation to us here to-night as among the men to whom our voice goes through the country, encouraging them to form dental clubs in the small cities. It is in this manner that the greater value of the paper will be found.

*Dr. F. Milton Smith.*—The thought to which our chairman has just given expression has been uppermost in my mind during the reading of the paper. We are fortunate in that the proceedings of our meetings go round the world. Papers of this character, if carefully and thoughtfully read, will greatly help the dental profession. It is meeting in this informal way that is going to make better men and better practitioners. I think I feel strongly upon this point because of the great help I have had from the little gatherings I have been permitted to enjoy in this town, perhaps at somebody's dinner-table or out for a walk with some friend.

*The President.*—Before we adjourn, I wish, in the name of the society, to tender, especially to our two medical friends and to Dr. Canaday, who has come to us from Albany, the most sincere thanks of the Institute for their kindness in addressing us to-night.

Adjourned.

FRED. L. BOGUE, M.D., D.D.S.,
*Editor The New York Institute of Stomatology.*

OFFICERS ELECTED.

THE annual meeting of the Institute was held on Tuesday afternoon, December 6, 1904, at the "Chelsea," No. 222 West Twenty-third Street, New York, the President, Dr. A. H. Brockway, in the chair.

The following officers were elected for 1905:

President, C. O. Kimball; Vice-President, S. E. Davenport; Recording Secretary, H. L. Wheeler; Corresponding Secretary, J. Buckley Locherty; Treasurer, J. Morgan Howe; Curator, S. H. McNaughton; Editor, F. L. Bogue.

*Executive Committee.*—F. Milton Smith, Chairman; C. F. Allan, E. A. Bogue.

